# Supplementary material for: CMYA5 establishes cardiac dyad architecture and positioning
Source: Nat Commun. 2022 Apr 21;13:2185. doi: 10.1038/s41467-022-29902-4 (PMC9023524; doi:10.1038/s41467-022-29902-4)
Supplement: Supplementary file 1 — Supplementary Information [file 41467_2022_29902_MOESM1_ESM.pdf]

Supplementary Information:
CMYA5 establishes cardiac dyad architecture positioning

Lu F, Ma Q, Xie W, Liou CL, Zhang D, Sweat ME, Jardin BD, Naya FJ, Guo Y, Cheng H, Pu WT

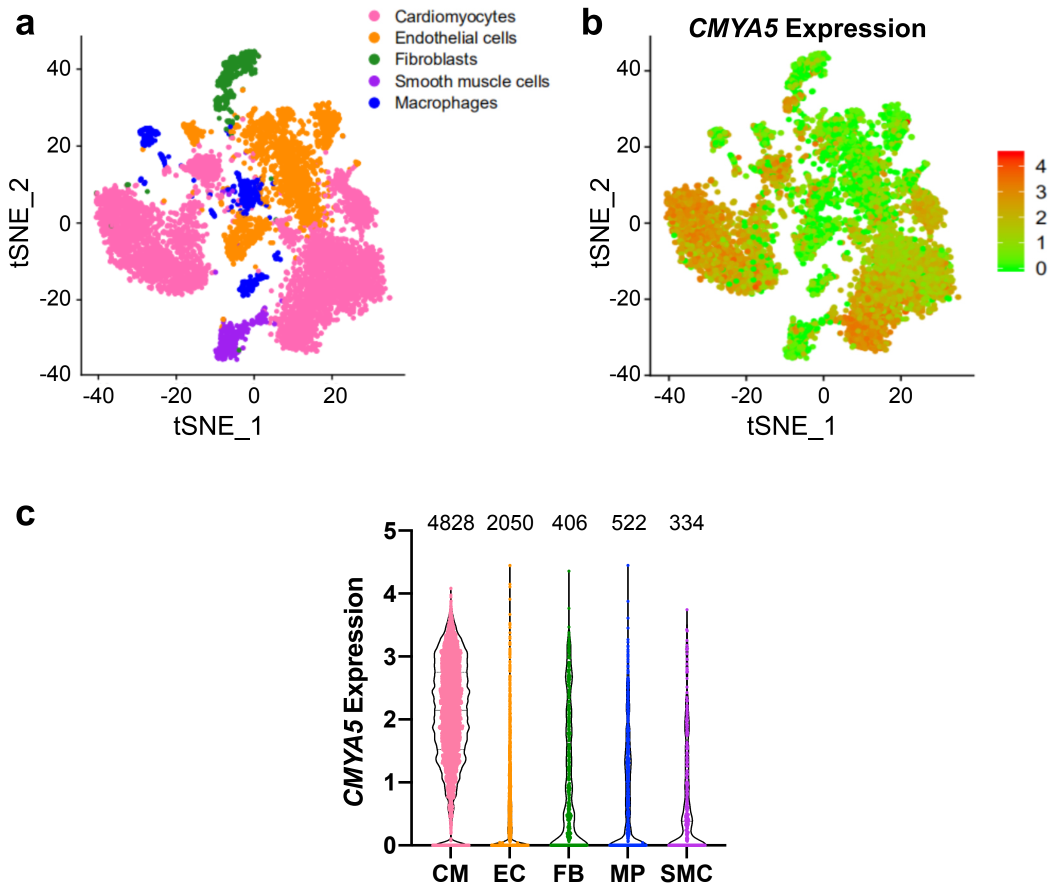

Supplementary Figure 1. Cardiac expression of *CMYA5*. Single cell RNA-seq of human heart from GSE109816. **a.** tSNE clustering of cardiac cells. Cardiomyocytes are shown in pink. **b.** *CMYA5* expression mapped onto tSNE clusters. **c.** Violin plot of *CMYA5* expression in major cardiac cell types, showing selective expression in cardiomyocytes (CM). EC, endothelial cell. FB, fibroblast. MP, macrophage. SMC, smooth muscle cells.

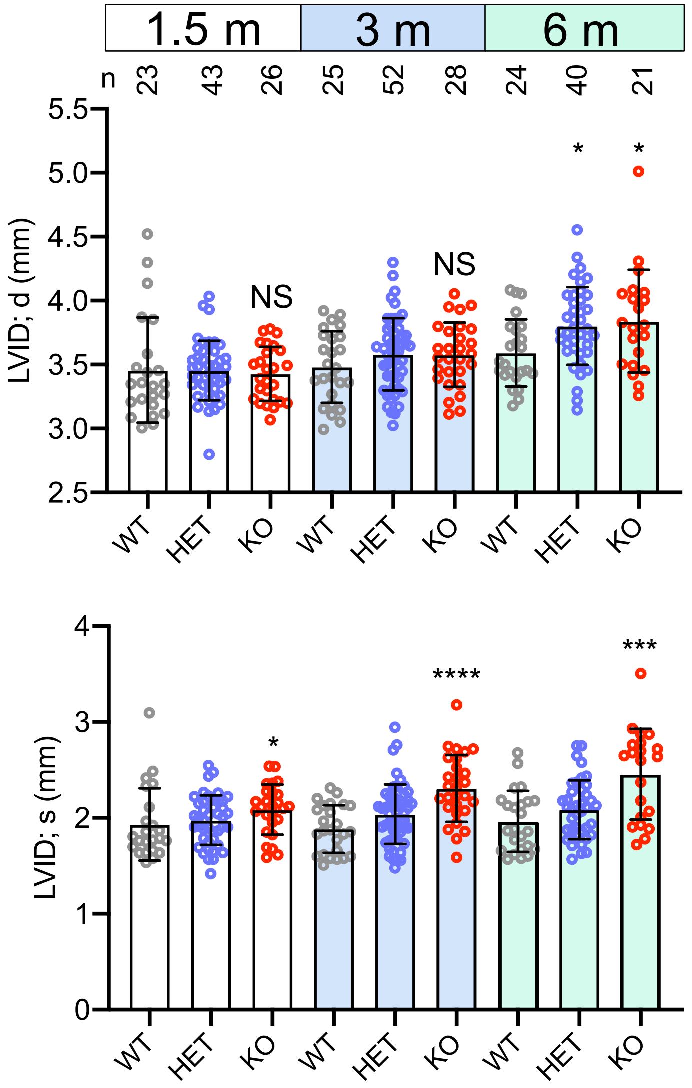

Supplementary Figure 2. Progressive left ventricular dilatation in *Cmya5* KO. Left ventricular internal diameter was measured in diastole (LVID;d) or systole (LVID;s). There was progressive LV dilatation in *Cmya5* KO but not *Cmya5* Het. Kruskal-Wallis with Dunn's multiple comparsion test vs. WT at the same time point. *, $P<0.05$; **, $P<0.01$; ****, $P<0.0001$.

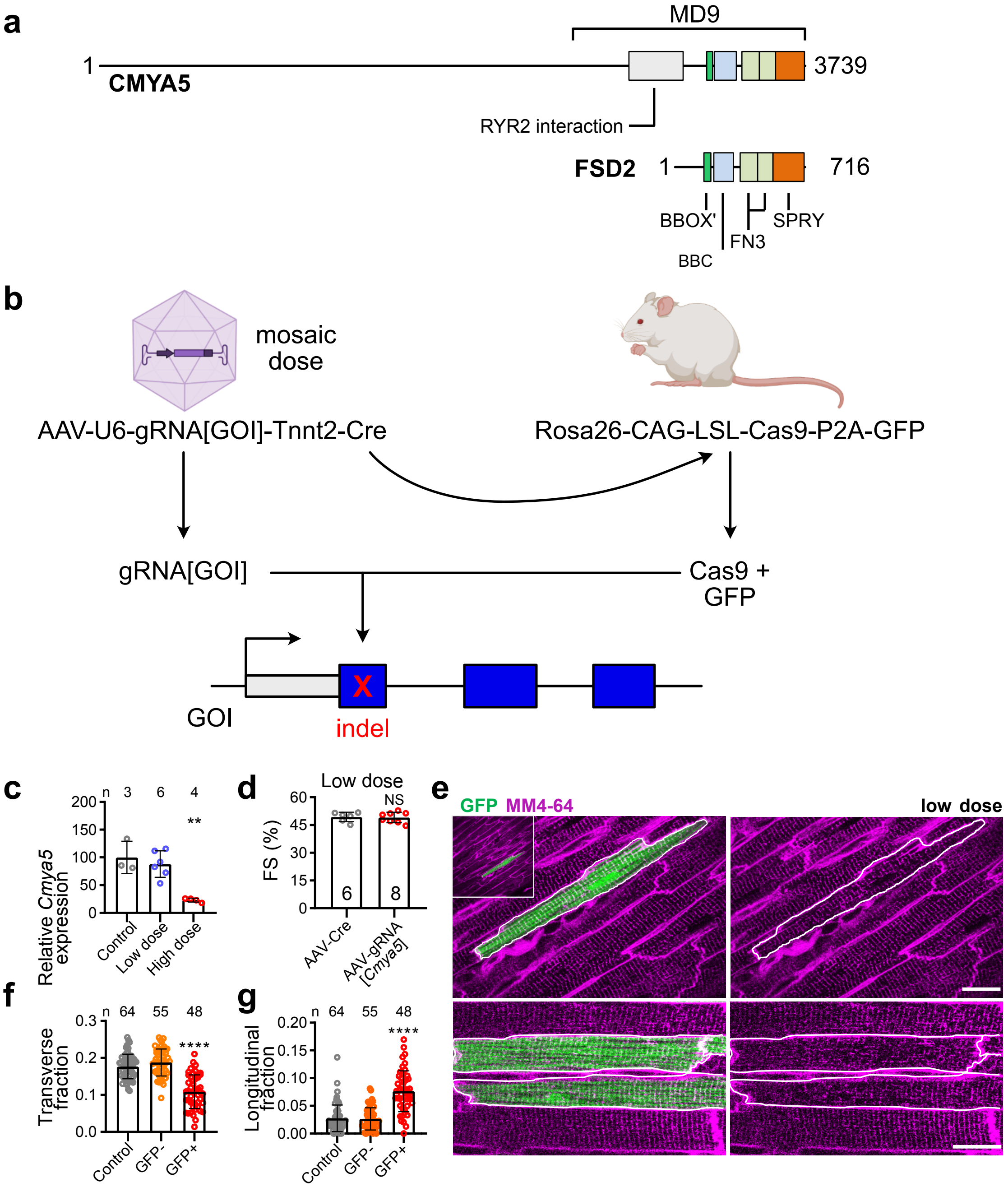

Supplementary Figure 3. CMYA5 is required cell autonomously for T-tubule maintenance. **a.** Schematic diagram of CMYA5 and FSD2 domain structure. Annotated domains are shown as rectangles. The C-terminus of CMYA5 is closely related in sequence, domains, and domain order to FSD2. The C-terminal region of CMYA5 named MD9 contains all the known interactions between CMYA5 and other proteins. **b.** CASAAV somatic mutagenesis strategy. AAV that expresses cardiomyocyte specific Cre and guide RNA targeting a mouse gene is delivered to Rosa26-fsCas9-GFP mice. GFP marks Cas9 expressing cells. Cas9 in combination with gRNA generates indels in target genes and thereby inactivates them. Artwork from biorender.com. **c-g.** CASAAV targeting *Cmya5* was used to inactivate *Cmya5* in most cardiomyocytes (high dose, 5.5E10 vg/g) or in a low fraction of cardiomyocytes (low dose, 1E10 vg/g). The high dose strongly reduced *Cmya5* mRNA, whereas low dose did not significantly change *Cmya5* mRNA (c; ANOVA with Dunnett's multiple comparison test vs. control). Low dose CASAAV-gRNA[*Cmya5*] did not affect heart systolic function (d; *t*-test). Low dose CASAAV-gRNA[*Cmya5*] transduced cells (GFP+, white outline) had disorganized T-tubules (MM4-64 staining). Quantification of T-tubule staining in GFP+ cells after low dose CASAAV-gRNA[*Cmya5*] showed reduced transverse and increased longitudinal T-tubule fraction (f-g; Kruskal-Wallis with Dunn's multiple comparison test vs. control). Bar, 20 µm. Images in e are representative of the 48-64 cardiomyocytes quantified in f-g.

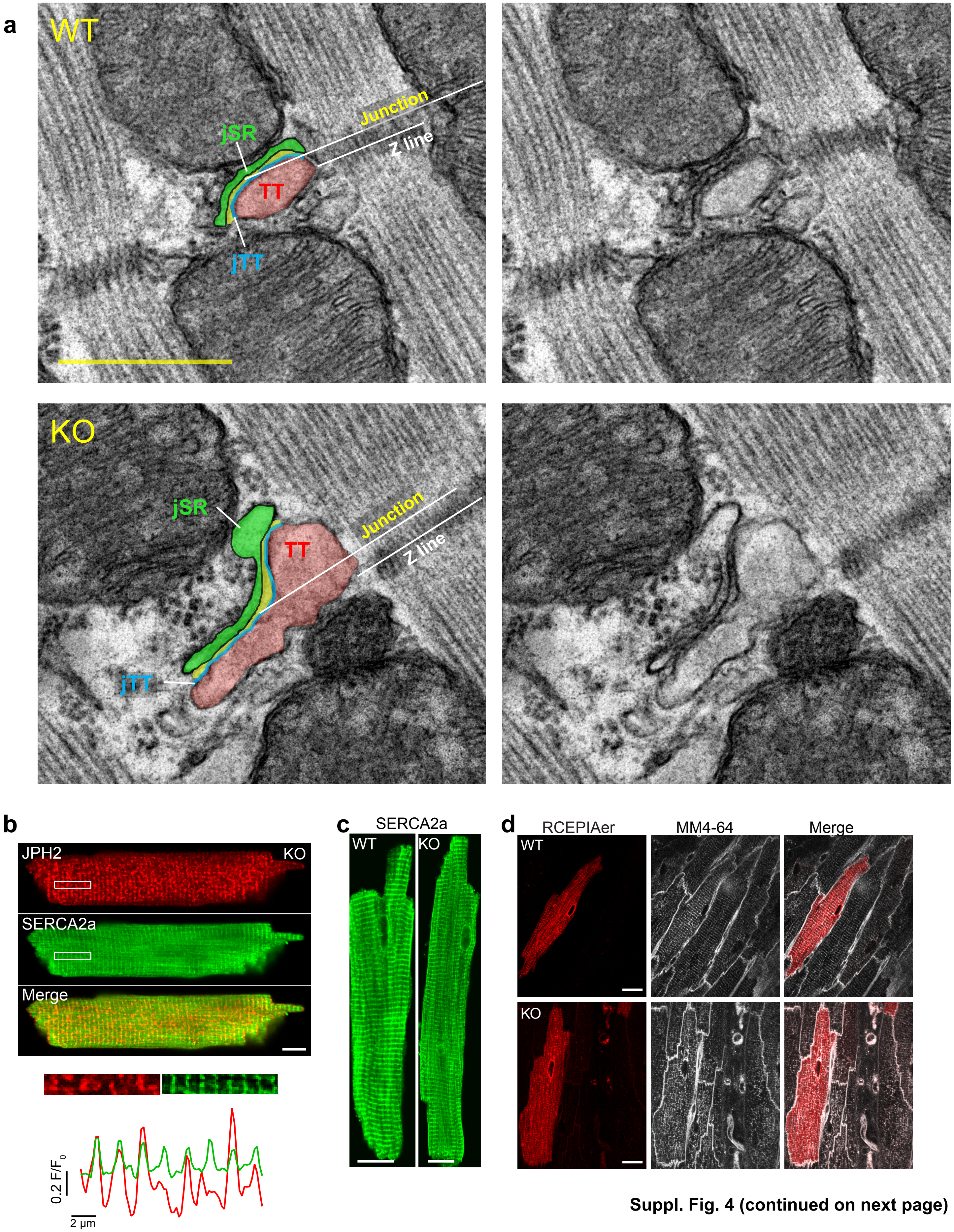

Suppl. Fig. 4 (continued on next page)

e

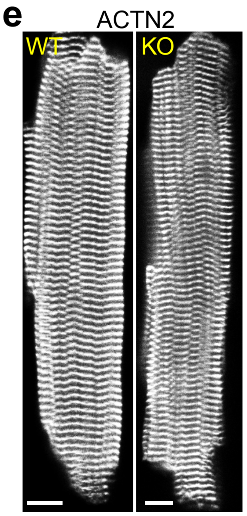

Supplementary Figure 4. Impact of Cmya5 KO on jSR, T-tubule, and ER organization. a. Transmission electron microscopy, demonstrating dyad architecture. Image on the right is shown with labels to the left. T-tubule (TT), junctional SR, (jSR), and junctional cleft (Junction; the ~12 nm gap between T-tubules and jSR) are shaded red, green, and yellow, respectively. Circularity is calculated as 4 * π * (area/perimeter^2). jTT, T-tubule boundary that abuts a junctional cleft. Coupling ratio is the jTT length divied by the TT perimeter. Junction-Z line distance is the length between the center of a junctional cleft and its adjacent Z-line, marked in the figure by white lines. Bar = 500 nm. **b.** Relationship of SERCA2a (ER) and jSR (JPH2) localization in KO cardiomyocytes. Boxed regions are enlarged to the right with plot of signals below. Bar = 10 µm. **c-d.** *Cmya5* KO did not impact overall localization of ER, marked by SERCA2a (c) or AAV-mediated expression of ER-localized fluorescent protein RCEPIAer (d). However, localization of MM4-64, marking T-tubules, was disrupted in KO. Bar = 15 µm. **e.** Localization of ACTN2, marking Z-lines, was not altered by *Cmya5* KO. Bar = 10 µm. Images in a-e are representative of 3 independent experiments.

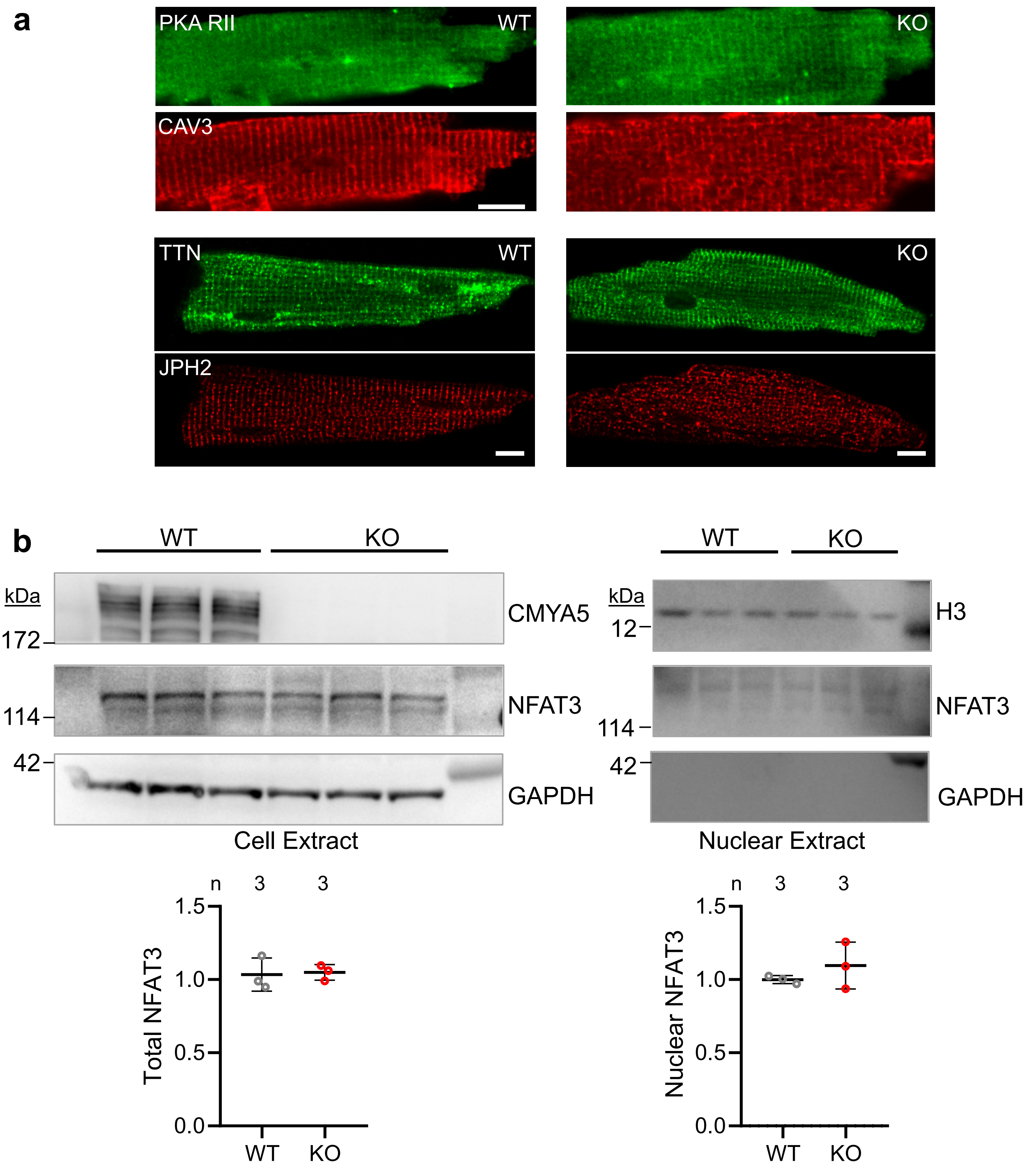

Suppl. Fig. 5, continued on next page.

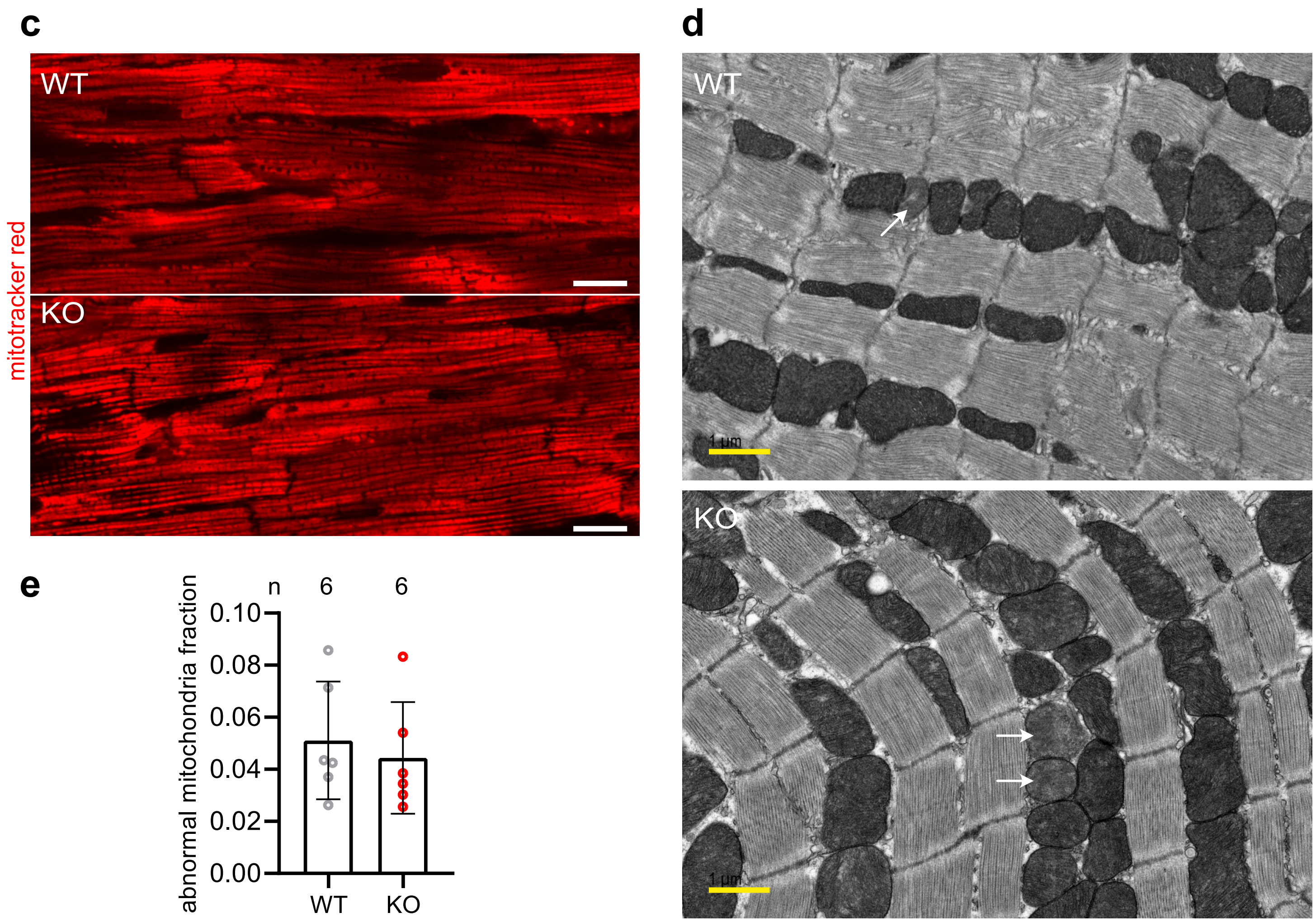

Supplementary Figure 5. Additional characterization of *Cmya5* KO cardiomyocytes. a. *Cmya5* WT and KO isolated adult cardiomyocytes immunostained for PKA subunit RIIA, T-tubule marker CAV3, TTN, or jSR marker JPH2. PKA and TTN localization were not disrupted in *Cmya5* KO cardiomyocytes. Bar = 10 µm. Representative of 3 independent experiments. **b**. Nuclear localization of NFAT3, a downstream target of PKA-CN signaling. Cell and nuclear extracts were prepared from control and *Cmya5* KO heart ventricles at 3 months of age. Proteins were detected by western blotting. We did not detect alteration of total or nuclear NFAT3 in *Cmya5* KO hearts. **c-e**. Mitochondrial organization and morphology in *Cmya5* WT and KO hearts. Freshly isolated hearts were perfused with mitotracker red and imaged using a confocal microscope (c). Overall mitochondrial organization was similar between genotypes. Bar = 10 µm. Transmission electron microscopy (d) likewise did not detect a difference in mitochondrial morphology between genotypes. Bar = 1 µm. Arrows point to examples of mitochondria classified as abnormal. Quantification of the fraction of abnormal mitochondria in transmission electron micrographs did not detect a difference between genoypes (e). Two images from each of three animals were quantified. Data are presented as mean ± SD.

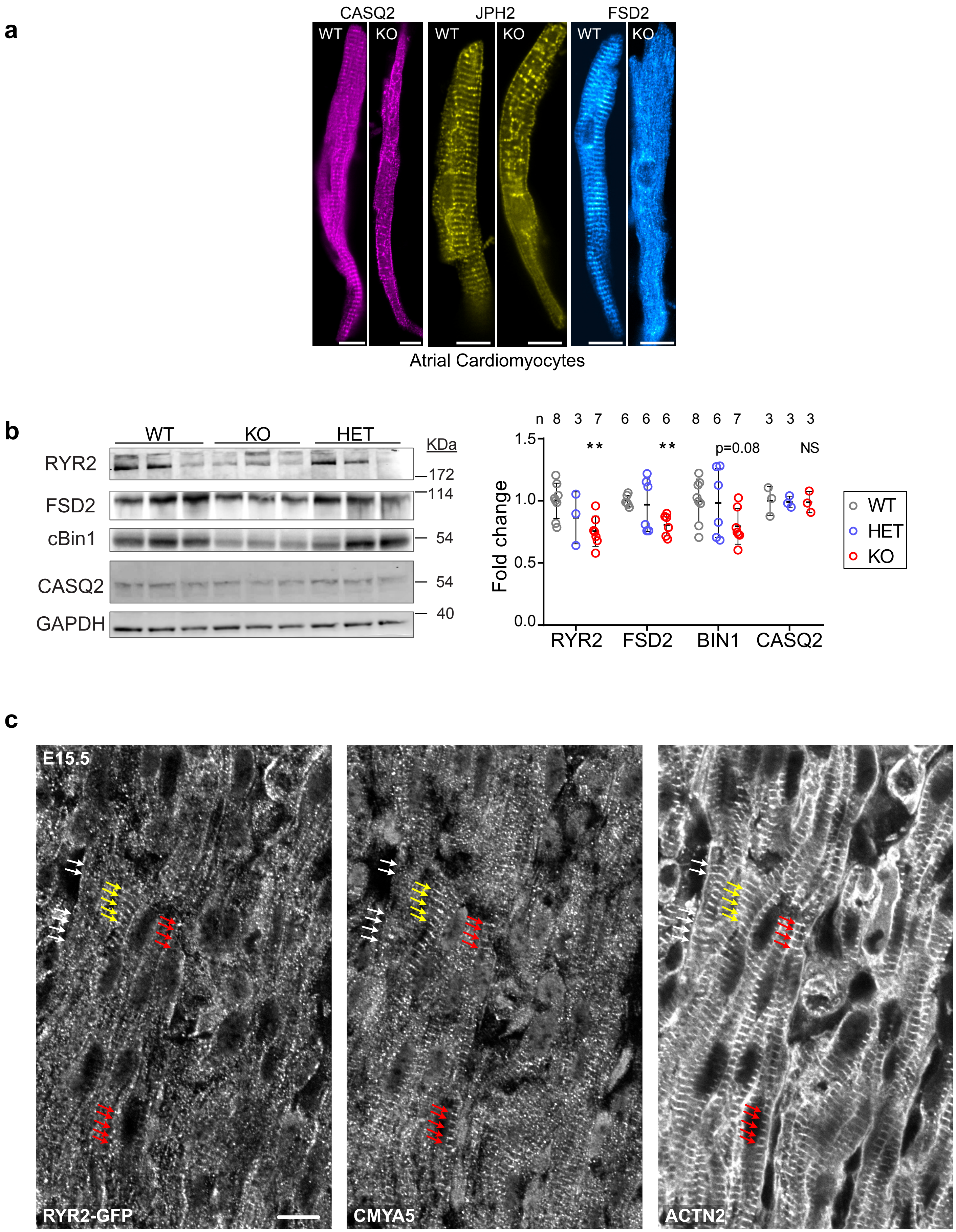

Suppl. Fig. 6. (continued on next page)

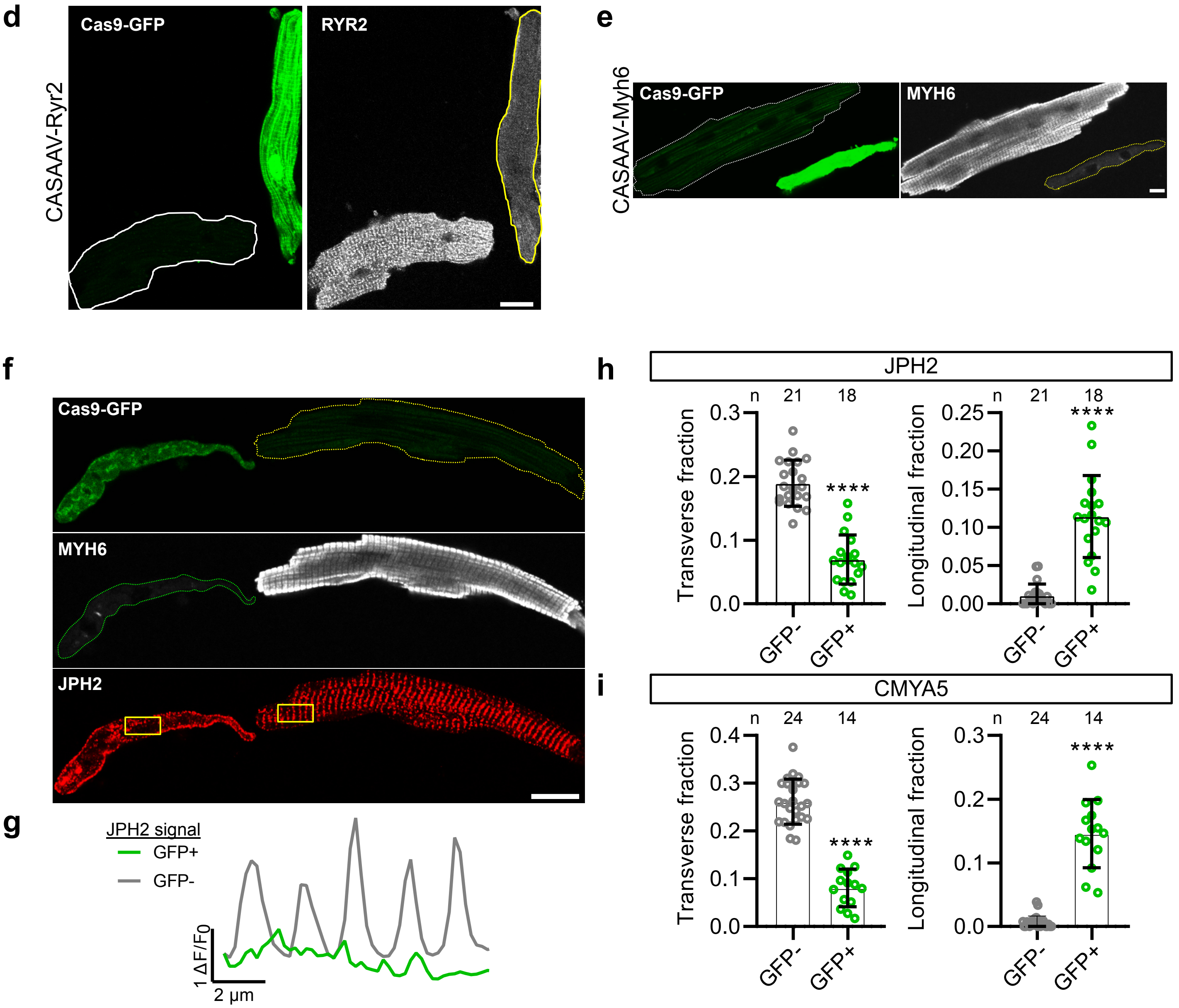

Supplementary Figure 6. Hierarchical relationship of CMYA5, sarcomeres, jSR, and T-tubules. a. Mature atrial cardiomyocytes immunostained for CASQ2, JPH2, and FSD2. Bar, 10 μm. Representative of 4 independent experiments. **b.** Western blot measuring the effect of *Cmya5* knockout or heterozygous mutation on protein levels of jSR (RYR2, FSD2, CASQ2) and T-tubule (cBIN1) components in 3 month-old heart lysates. Western quantification is shown to the right. ANOVA with Dunnett's multiple comparison test vs. WT. **c.** E15.5 ventricular myocardial sections from RYR2-GFP embryos, stained for GFP, CMYA5, or ACTN2. White arrows, ACTN2 without co-localized CMYA5 or RYR2-GFP; yellow arrows, Bar, 10 μm. Representative of 4 independent experiments. **d.** Validation of CASAAV somatic mutagenesis depletion of RYR2 in GFP+ cardiomyocytes. See also Guo et al., Circ. Res., 2017. Representative of 3 independent experiments. **e.** Validation of CASAAV depletion of MYH6 in GFP+ cardiomyocytes. See also Guo et al., Nat. Commun., 2018. Representative of 3 independent experiments. **f-i.** Quantification of jSR organization in cardiomyocytes after CASAAV-Myh6 treatment. JPH2 signal in boxed regions in (f) is plotted in (g). Quantification of T-tubule organization based on JPH2 signal. Mann-Whitney test. n, number of cardiomyocytes. **h.** Quantification of CMYA5 organization in cardiomyocytes after CASAAV-Myh6 treatment. Mann-Whitney test. Data are presented as mean ± SD. NS, not significant. **, P<0.01; ****, P<0.0001.

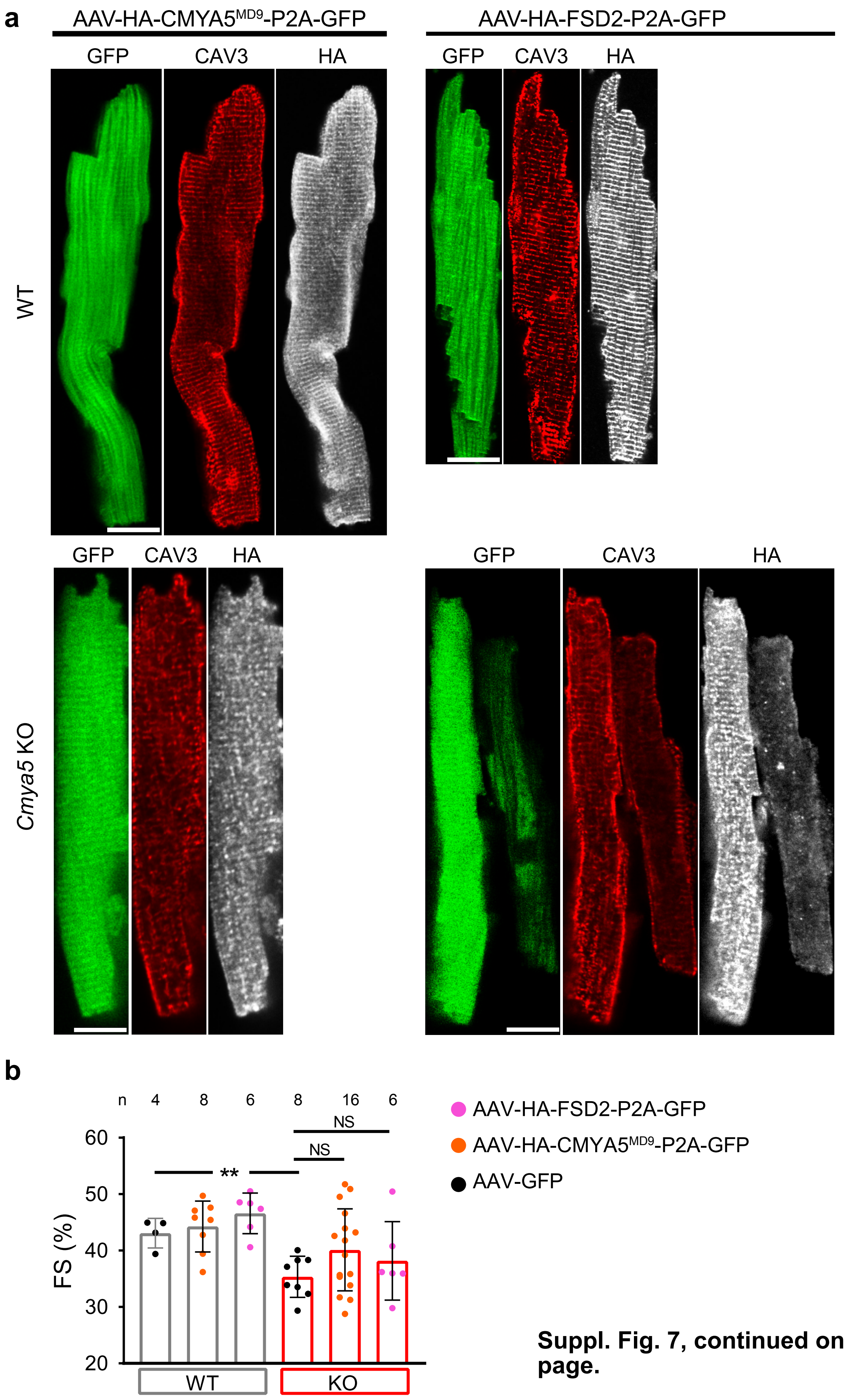

Suppl. Fig. 7, continued on next page.

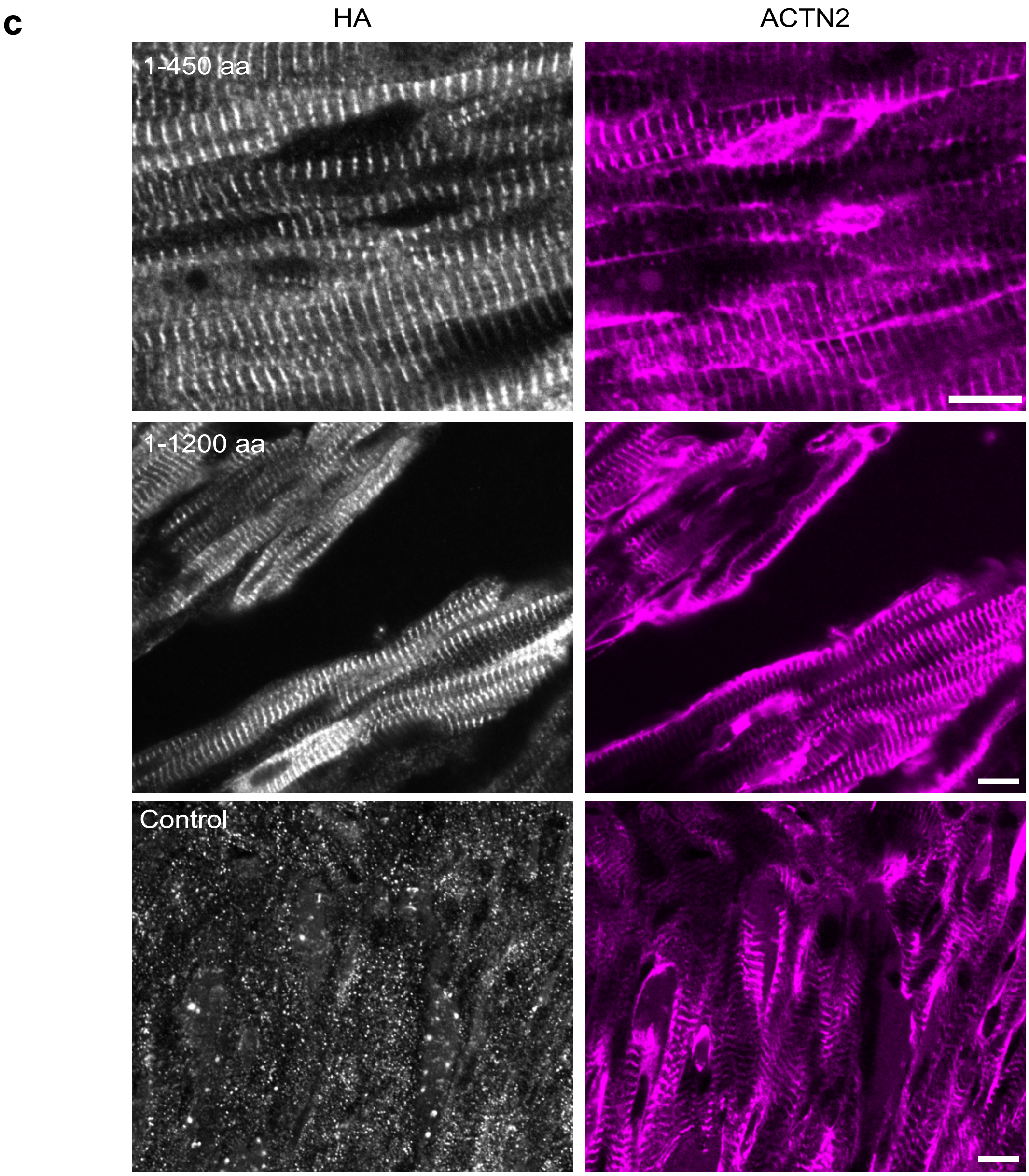

Supplementary Figure 7. CMYA5^{MD9} and FSD2 failed to complement *Cmya5* knockout. The MD9 fragment of CMYA5 (CMYA5^{MD9}) or FSD2 were expressed in *Cmya5* knockout or control hearts using AAV9 and the cardiac troponin T promoter. **a.** CMYA5^{MD9} and FSD2 localized in a striated pattern and co-localized with T-tubules in WT. CMYA5^{MD9} and FSD2 did not localize normally in *Cmya5* KO, and they did not rescue their T-tubule defects. Bar, 20 µm. Representative of 4 independent experiments. **b.** The contractile defect of *Cmya5* KO hearts was not rescued by CMYA5^{MD9} or FSD2 overexpression. ANOVA with Dunnett's multiple comparison test vs control treatment within each genotype. **, P<0.01. NS, not significant. Data are presented as mean ± SD. **c.** Localization of CMYA5 N-terminal fracments in cardiomyocytes in vivo. AAV that expressed HA-CMYA5[1-450] or HA-CMYA5[1-1200] were administered to newborn mice. Distribution of HA-tagged proteins was determined by immunostaining at P7 and compared to ACTN2 staining of Z-lines. Bar = 10 µm. Representative of 4 independent experiments.

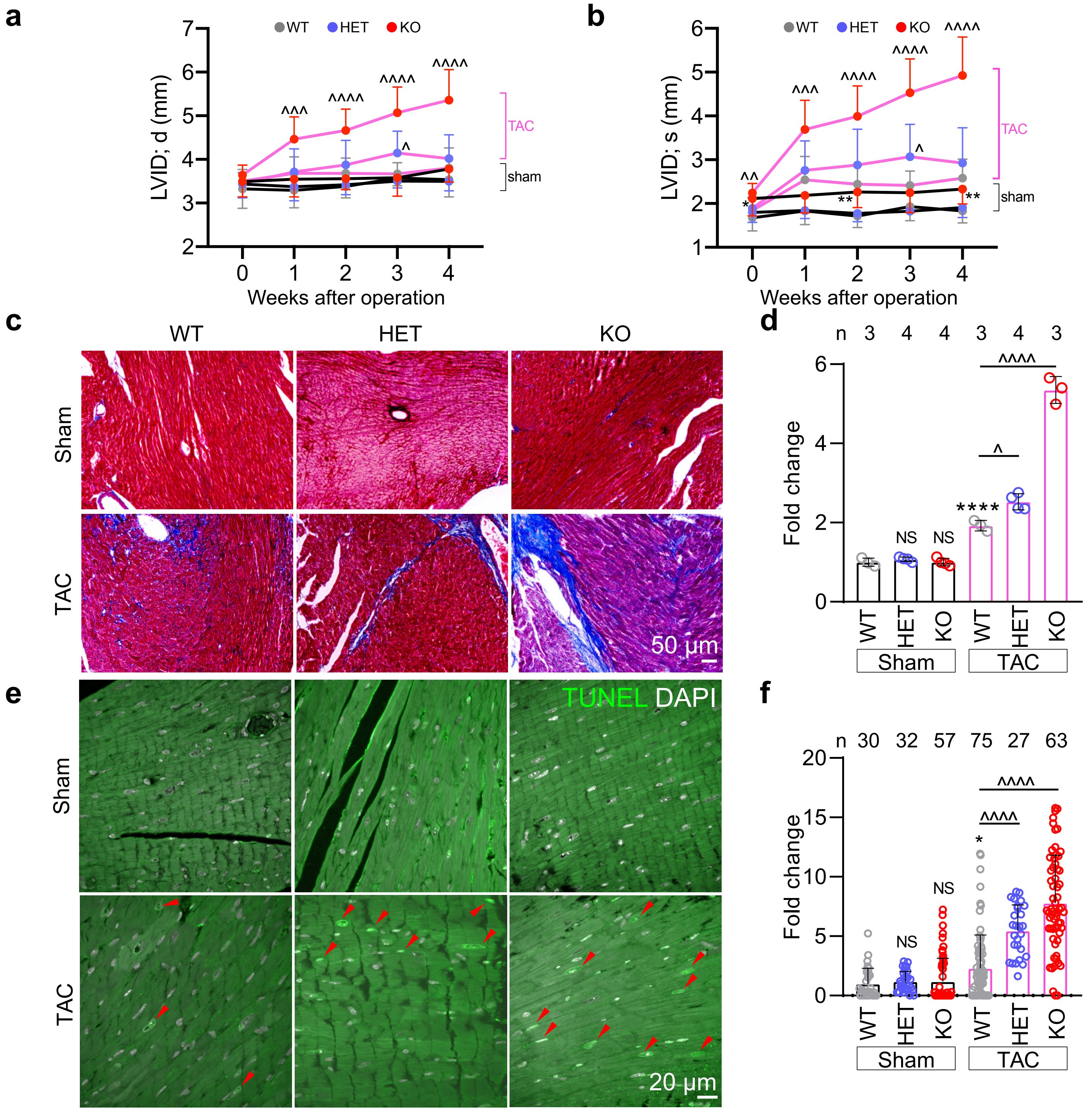

Supplementary Figure 8. Cardiac remodeling, fibrosis, and apoptosis in *Cmya5* KO under pressure overload. a-b. Cardiac remodeling caused by *Cmya5* KO and TAC. Left ventricular dimensions were measured by echocardiography at the indicated time points after TAC or Sham surgery. *Cmya5* KO mice developed LV dilatation. Repeated measures two-way ANOVA with Dunnett's multiple comparisons test vs. WT within sham (*) or TAC (^) groups. n=5 mice per group. **c-d.** Cardiac fibrosis in *Cmya5* KO. Heart sections were stained with Masson's trichrome. Blue staining indicates fibrotic tissue. d, Fold-change in the area of myocardial sections occupied by fibrotic tissue. ANOVA with Dunnett's multiple comparison test versus WT-Sham (*) or WT-TAC (^). n, number of mice. One short axis section through the ventricles was analyzed per animal. Representative of 3 to 4 independent experiments. **e-f.** TUNEL staining of myocardial sections. Red arrowheads point out examples of apoptotic cells. f, Fold-change in the number of apoptotic cells per field. Kruskal-Wallis with Dunn's multiple comparison stest versus WT-Sham (*) or WT-TAC (^). n, number of fields. Representative of at least 10 fields were analyzed per mouse. NS, not significant. * or ^, P<0.05. ** or ^^, P<0.01. ^^^, P<0.001. **** or ^^^^, P<0.0001. Data are presented as mean ± SD.

Supplementary Table 1. Oligonucleotides used in this study

Genotyping primers

Genotype	primer1	primer2	common primer
Cmya5Δ	AGGCTTCACCTTGCTTTGATAG	TGTGAAAGCAAACCCAATATC	TTCACCATAGGTTATCAGATGAGG
RosaCas9gfp/Cas9gfp	CCGAAAATCTGTGGGAAGTC	CCAAGTGGGCAGTTTACCGTAAATAC	AAGGGAGCTGCAGTGGAGTA
Ryr2-gfp	ATATCACTCCTAGACATACCCTCA	CTTCAGCTCGATGCGGTTCAC	AGACCAGACAAGCCATCACACTA

qPCR primers

Gene	primer1	primer2
Gapdh	GGATAAACCTGCCAACTATGATGAC	GTAGCCCAGGATGCCCTTTAGT
Cmya5 pair1	AAATGGACAAGGCACTGGAC	CTCATCATACTGCGCCAAAA
Cmya5 pair2	GTTAACCTCCCTCCCAACGACAATTACTT	CCGTCCCGTTCGCGGAGATCTGGAG

gRNAs for CRISPR/Cas9 mutagenesis

Gene target	gRNA target 1	gRNA target 2
Cmya5	GTTATCAGATGAGGATGAGG	CGAGGTTTCAGCACTCGACA
Ryr2	TGAGGTGGTTCTGCAGTGCA	TGCTTGGCAGCAGAAGGATT
Myh6	CAGGCACGAAGCACTCCGTG	CGCCCAGATGGCTGACTTCG

Supplementary Table 2. Antibodies used in this study

Antibody	Source	Catalog No.	Host species	Western Blotting dilution	Immunofluorescence dilution
CMYA5	Dr. Francisco J. Naya, Boston University	-	Rabbit	1:500	1:100
RYR2	Sigma-Aldrich	R128	Mouse	1:500	1:100
CAV3	Life Technologies	PA1066	Rabbit	-	1:100
CASQ2	Abcam	ab3516	Rabbit	1:500	1:100
JPH2	Invitrogen	40-5300	Rabbit	-	1:100
FSD2	Santa Cruz Biotechnology	sc-393072	Mouse	1:500	1:100
SAA	Sigma-Aldrich	A7811	Mouse	-	1:100
BIN1	Rockland Immunochemicals	200-301-E63	Mouse	1:500	-
GAPDH	Proteintech	60004-1-Ig	Mouse	1:500	-
SERCA2a	Invitrogen	MA3-919	Mouse	-	1:100
HA tag	Cell Signaling Technology	3724S	Rabbit	-	1:100
HA tag	BioLegend	901513	Mouse	-	1:100
MYH6	Developmental Studies Hybridoma Bank	A4.1025	Mouse	-	1:100
NFAT3	Santa Cruz Biotechnology	sc-1153	Goat	1:500	-
Histone H3	Abcam	ab1791	Rabbit	1:500	-
Titin	Developmental Studies Hybridoma Bank	9D10	Mouse	-	1:100
PKA	BD Biosciences	612242	Mouse	-	1:100
Streptavidin-Horseradish Peroxidase (HRP) Conjugate	Invitrogen	SA10001	-	1:3000	-
Donkey-anti-Mouse HRP	Rockland Immunochemicals	610-703-124		1:3000	-
Donkey-anti-Rabbit HRP	Rockland Immunochemicals	611-703-127		1:3000	-
Donkey-anti-Mouse 488	Invitrogen	A32766		-	1:200
Donkey-anti-Rabbit 488	Invitrogen	A32790		-	1:200
Donkey-anti-Mouse 555	Invitrogen	A31570		-	1:200
Donkey-anti-Rabbit 555	Invitrogen	A31572		-	1:200
Donkey-anti-Mouse 647	Invitrogen	A31571		-	1:200
Donkey-anti-Rabbit 647	Invitrogen	A31573		-	1:200
