# Supplementary material for: CMYA5 establishes cardiac dyad architecture and positioning
Source: Nat Commun. 2022 Apr 21;13:2185. doi: 10.1038/s41467-022-29902-4 (PMC9023524; doi:10.1038/s41467-022-29902-4)
Supplement: Supplementary file 3 — Description of Additional Supplementary Files [file 41467_2022_29902_MOESM3_ESM.docx]

**Description of Additional Supplementary Files**

File Name: Supplementary Data 1

Description: BioID mass spect analysis of biotinylated proteins from hearts treated with BioID-Junctin, BioID-Triadin, and GFP. Proteins were purified by binding to streptavidin and analyzed by liquid chromatography with tandem mass spectrometry. Proteins are rank ordered by the average signal in the BioID samples. BJ, BioID-Junctin. BT, BioID-Triadin.
