# Supplementary material for: CMYA5 establishes cardiac dyad architecture and positioning
Source: Nat Commun. 2022 Apr 21;13:2185. doi: 10.1038/s41467-022-29902-4 (PMC9023524; doi:10.1038/s41467-022-29902-4)
Supplement: Supplementary file 5 — Reporting Summary [file 41467_2022_29902_MOESM5_ESM.pdf]

nature portfolio

Corresponding author(s): William T. Pu

Last updated by author(s): 2022-2-20

Reporting Summary

Nature Portfolio wishes to improve the reproducibility of the work that we publish. This form provides structure for consistency and transparency in reporting. For further information on Nature Portfolio policies, see our Editorial Policies and the Editorial Policy Checklist.

Statistics

For all statistical analyses, confirm that the following items are present in the figure legend, table legend, main text, or Methods section.

n/a	Confirmed	
☐	☒	The exact sample size (n) for each experimental group/condition, given as a discrete number and unit of measurement
☐	☒	A statement on whether measurements were taken from distinct samples or whether the same sample was measured repeatedly
☐	☒	The statistical test(s) used AND whether they are one- or two-sided<br>*Only common tests should be described solely by name; describe more complex techniques in the Methods section.*
☒	☐	A description of all covariates tested
☒	☐	A description of any assumptions or corrections, such as tests of normality and adjustment for multiple comparisons
☐	☒	A full description of the statistical parameters including central tendency (e.g. means) or other basic estimates (e.g. regression coefficient) AND variation (e.g. standard deviation) or associated estimates of uncertainty (e.g. confidence intervals)
☐	☒	For null hypothesis testing, the test statistic (e.g. F, t, r) with confidence intervals, effect sizes, degrees of freedom and P value noted<br>*Give P values as exact values whenever suitable.*
☒	☐	For Bayesian analysis, information on the choice of priors and Markov chain Monte Carlo settings
☒	☐	For hierarchical and complex designs, identification of the appropriate level for tests and full reporting of outcomes
☒	☐	Estimates of effect sizes (e.g. Cohen's d, Pearson's r), indicating how they were calculated

Our web collection on statistics for biologists contains articles on many of the points above.

Software and code

Policy information about availability of computer code

Data collection	VisualSonics Vevo 2100 for echocardiogram, Olympus FV3000 confocal microscope for imaging, Thermo Fisher Scientific LTQ Orbitrap Velos Elite ion-trap mass spectrometer
Data analysis	Graphpad Prism 9, Fiji (ImageJ 1.51w), Interactive Data Language 7.1, SEQUEST™ HT

For manuscripts utilizing custom algorithms or software that are central to the research but not yet described in published literature, software must be made available to editors and reviewers. We strongly encourage code deposition in a community repository (e.g. GitHub). See the Nature Portfolio guidelines for submitting code & software for further information.

Data

Policy information about availability of data

All manuscripts must include a data availability statement. This statement should provide the following information, where applicable:

- Accession codes, unique identifiers, or web links for publicly available datasets
- A description of any restrictions on data availability
- For clinical datasets or third party data, please ensure that the statement adheres to our policy

The authors declare that the data supporting the findings of this paper are available within the paper, its supplemental figures and tables. The proteomics data has been deposited to PRIDE, PXD028960. Single cell RNA-seq data on normal human hearts was obtained from GSE109816 (https://www.ncbi.nlm.nih.gov/geo/query/acc.cgi?acc=GSE109816)

Field-specific reporting

Please select the one below that is the best fit for your research. If you are not sure, read the appropriate sections before making your selection.

- [x] Life sciences
- [ ] Behavioural & social sciences
- [ ] Ecological, evolutionary & environmental sciences

For a reference copy of the document with all sections, see nature.com/documents/nr-reporting-summary-flat.pdf

Life sciences study design

All studies must disclose on these points even when the disclosure is negative.

Sample size	Power calculations were performed for physiological measurements of organ function. Sample sizes exceeded the number of samples determined by power calculations, which were based on biologically meaningful effect size and our historical standard deviations.
Data exclusions	No data were excluded from analysis. For live cardiomyocyte imaging, the round globe cells were not studied as they were undergoing dying process.
Replication	Biological replicates for each figure panel is now provided in the figure legend.
Randomization	Samples were randomly allocated.
Blinding	The investigators were blinded to group allocation during data acquisition and analysis. See also Methods.

Reporting for specific materials, systems and methods

We require information from authors about some types of materials, experimental systems and methods used in many studies. Here, indicate whether each material, system or method listed is relevant to your study. If you are not sure if a list item applies to your research, read the appropriate section before selecting a response.

Materials & experimental systems

n/a	Involved in the study	
[ ]	[x]	Antibodies
[ ]	[x]	Eukaryotic cell lines
[x]	[ ]	Palaeontology and archaeology
[ ]	[x]	Animals and other organisms
[x]	[ ]	Human research participants
[x]	[ ]	Clinical data
[x]	[ ]	Dual use research of concern

Methods

n/a	Involved in the study	
[x]	[ ]	ChIP-seq
[x]	[ ]	Flow cytometry
[x]	[ ]	MRI-based neuroimaging

Antibodies

Antibodies used

Additional information for all the antibodies used are provided in supplemental tables.
CMYA5 Dr. Francisco J. Naya, Boston University Rabbit 1:500 (WB) 1:100(IF)
RYR2 Sigma-Aldrich R128 Mouse 1:500 (WB) 1:100 (IF)
CAV3 Life Technologies PA1066 Rabbit 1:100 (IF)
CASQ2 Abcam ab3516 Rabbit 1:500 (WB) 1:100 (IF)
JPH2 Invitrogen 40-5300 Rabbit 1:100 (IF)
FSD2 Santa Cruz Biotechnology sc-393072 Mouse 1:500 (WB) 1:100 (IF)
SAA Sigma-Aldrich A7811 Mouse 1:100 (IF)
BIN1 Rockland Immunochemicals 200-301-E63 Mouse 1:500 (WB)
GAPDH Proteintech 60004-1-Ig Mouse 1:500 (WB)
SERCA2a Invitrogen MA3-919 Mouse 1:100 (IF)
HA tag Cell Signaling Technology 3724S Rabbit 1:100 (IF)
HA tag BioLegend 901513 Mouse 1:100 (IF)
MYH6 Developmental Studies Hybridoma Bank A4.1025 Mouse 1:100 (IF)
NFAT3 Santa Cruz Biotechnology sc-1153 Goat 1:500 (WB)
Histone H3 Abcam ab1791 Rabbit 1:500 (WB)
Titin Developmental Studies Hybridoma Bank 9D10 Mouse 1:100 (IF)
PKA BD Biosciences 612242 Mouse 1:100 (IF)
Streptavidin-Horseradish Peroxidase (HRP) Conjugate Invitrogen SA10001 1:3000 (WB)

Validation

CMYA5 antibody -validated by multiple papers: 1(IF), 2(WB)

RYR2 antibody -validated by multiple papers: 3(IF) 4(IF) 5(WB)
CAV3 antibody -validated by multiple papers: 6(IF) 7(IF)
CASQ2 antibody -validated by multiple papers: 8(WB) 9(WB, IF)
JPH2 antibody -validated by multiple papers: 10(IF) 11(IF)
FSD2 antibody -validated by Santa Cruz Biotechnology: 12 (IF, WB)
SAA antibody -validated by multiple papers: 13(IF) 14(IF)
BIN1 antibody -validated by multiple papers: 10(WB) 15(WB)
GAPDH antibody -validated by paper: 16 (WB)
SERCA2a antibody -validated by multiple papers: 17(IF) 18(IF)
HA tag antibody -validated by multiple papers: 19(IF) 20(IF)
HA tag antibody -validated by multiple papers: 21(IF) 22(IF)
MYH6 antibody -validated by multiple papers: 23(IF) 24(IF)
NFAT3 antibody -validated by multiple papers: 25(WB) 26(WB)
Histone H3 antibody -validated by multiple papers: 27(WB) 28(WB)
Titin antibody -validated by multiple papers: 29(IF) 30(IF)
PKA antibody -validated by multiple papers: 31(IF) 32(IF)
Streptavidin-Horseradish Peroxidase (HRP) Conjugate antibody -validated by multiple papers: 33(WB) 34(WB)

Citations

1. Durham, J. T. et al. Myospryn Is a Direct Transcriptional Target for MEF2A That Encodes a Striated Muscle, α-Actinin-interacting, Costamere-localized Protein*. J. Biol. Chem. 281, 6841–6849 (2006).
2. Kielbasa, O. M. et al. Myospryn is a calcineurin-interacting protein that negatively modulates slow-fiber-type transformation and skeletal muscle regeneration. FASEB J. 25, 2276–2286 (2011).
3. Shang, W. et al. Imaging Ca2+ nanosparks in heart with a new targeted biosensor. Circ. Res. 114, 412–420 (2014).
4. Sahu, G. et al. Junctophilin Proteins Tether a Cav1-RyR2-KCa3.1 Tripartite Complex to Regulate Neuronal Excitability. Cell Rep. 28, 2427–2442.e6 (2019).
5. Vaithianathan, T. et al. Subtype identification and functional characterization of ryanodine receptors in rat cerebral artery myocytes. American Journal of Physiology-Cell Physiology vol. 299 C264–C278 (2010).
6. Guo, Y. et al. Hierarchical and stage-specific regulation of murine cardiomyocyte maturation by serum response factor. Nat. Commun. 9, 3837 (2018).
7. VanDusen, N. J. et al. Massively parallel in vivo CRISPR screening identifies RNF20/40 as epigenetic regulators of cardiomyocyte maturation. Nat. Commun. 12, 4442 (2021).
8. Shankar, T. S. et al. Cardiac-specific deletion of voltage dependent anion channel 2 leads to dilated cardiomyopathy by altering calcium homeostasis. Nat. Commun. 12, 4583 (2021).
9. Feyen, D. A. M. et al. Metabolic Maturation Media Improve Physiological Function of Human iPSC-Derived Cardiomyocytes. Cell Rep. 32, 107925 (2020).
10. Guo, Y. et al. Analysis of Cardiac Myocyte Maturation Using CASAAV, a Platform for Rapid Dissection of Cardiac Myocyte Gene Function In Vivo. Circ. Res. 120, 1874–1888 (2017).
11. Munro, M. L. et al. Junctophilin-2 in the nanoscale organisation and functional signalling of ryanodine receptor clusters in cardiomyocytes. J. Cell Sci. 129, 4388–4398 (2016).
12. https://www.scbt.com/p/fsd2-antibody-h-11
13. Prondzynski, M. et al. Disease modeling of a mutation in α-actinin 2 guides clinical therapy in hypertrophic cardiomyopathy. EMBO Mol. Med. 11, e11115 (2019).
14. Chen, X. et al. QKI is a critical pre-mRNA alternative splicing regulator of cardiac myofibrillogenesis and contractile function. Nat. Commun. 12, 89 (2021).
15. An, S. et al. Adverse transverse-tubule remodeling in a rat model of heart failure is attenuated with low-dose triiodothyronine treatment. Mol. Med. 25, 53 (2019).
16. Leto, D. E. et al. Genome-wide CRISPR Analysis Identifies Substrate-Specific Conjugation Modules in ER-Associated Degradation. Mol. Cell 73, 377–389.e11 (2019).
17. Hu, L.-Y. R. et al. Deregulated Ca2+ cycling underlies the development of arrhythmia and heart disease due to mutant obscurin. Sci Adv 3, e1603081 (2017).
18. Callaghan, N. I. et al. Functional culture and in vitro genetic and small-molecule manipulation of adult mouse cardiomyocytes. Commun Biol 3, 229 (2020).
19. Tunc-Ozcan, E. et al. Activating newborn neurons suppresses depression and anxiety-like behaviors. Nat. Commun. 10, 3768 (2019).
20. Chai, Q. et al. A Mycobacterium tuberculosis surface protein recruits ubiquitin to trigger host xenophagy. Nature Communications vol. 10 (2019).
21. Bennett, B. D. et al. A furin-like convertase mediates propeptide cleavage of BACE, the Alzheimer's beta -secretase. J. Biol. Chem. 275, 37712–37717 (2000).
22. Liu, Z. et al. Autism-like behaviours and germline transmission in transgenic monkeys overexpressing MeCP2. Nature 530, 98–102 (2016).
23. Gabay Yehezkely, R. et al. Intracellular Role for the Matrix-Modifying Enzyme Lox in Regulating Transcription Factor Subcellular Localization and Activity in Muscle Regeneration. Dev. Cell 53, 406–417.e5 (2020).
24. O'Brien, J. H., Hernandez-Lagunas, L., Artinger, K. B. & Ford, H. L. MicroRNA-30a regulates zebrafish myogenesis through targeting the transcription factor Six1. J. Cell Sci. 127, 2291–2301 (2014).
25. Ding, B. et al. Temporal regulation of nuclear factor one occupancy by calcineurin/NFAT governs a voltage-sensitive developmental switch in late maturing neurons. J. Neurosci. 33, 2860–2872 (2013).
26. Nguyen, T. et al. NFAT-3 is a transcriptional repressor of the growth-associated protein 43 during neuronal maturation. J. Biol. Chem. 284, 18816–18823 (2009).
27. Kong, N. R. et al. Zinc Finger Protein SALL4 Functions through an AT-Rich Motif to Regulate Gene Expression. Cell Rep. 34, 108574 (2021).

28. Reader, J. et al. Multistage and transmission-blocking targeted antimalarials discovered from the open-source MMV Pandemic Response Box. Nat. Commun. 12, 269 (2021).
29. Ali, M. A. M. et al. Titin is a target of matrix metalloproteinase-2: implications in myocardial ischemia/reperfusion injury. Circulation 122, 2039–2047 (2010).
30. Molnár, I. et al. DAAM is required for thin filament formation and Sarcomerogenesis during muscle development in Drosophila. PLoS Genet. 10, e1004166 (2014).
31. Reynolds, J. G., McCalmon, S. A., Donaghey, J. A. & Naya, F. J. Deregulated protein kinase A signaling and myospryn expression in muscular dystrophy. J. Biol. Chem. 283, 8070–8074 (2008).
32. Reynolds, J. G., McCalmon, S. A., Tomczyk, T. & Naya, F. J. Identification and mapping of protein kinase A binding sites in the costameric protein myospryn. Biochim. Biophys. Acta 1773, 891–902 (2007).
33. Yap, K., Chung, T. H. & Makeyev, E. V. Hybridization-proximity labeling reveals spatially ordered interactions of nuclear RNA compartments. Molecular Cell vol. 82 463–478.e11 (2022).
34. Cardamone, M. D. et al. Neuralized-like protein 4 (NEURL4) mediates ADP-ribosylation of mitochondrial proteins. J. Cell Biol. 221, (2022).

Eukaryotic cell lines

Policy information about cell lines

Cell line source(s)	HEK293T cells (human, ATCC, CRL-3216) for virus production
Authentication	Cell line is authenticated by ATCC via STR profiling
Mycoplasma contamination	The cell line was tested negative for mycoplasma
Commonly misidentified lines (See ICLAC register)	No commonly misidentified cell lines were used in the study

Animals and other organisms

Policy information about studies involving animals; ARRIVE guidelines recommended for reporting animal research

Laboratory animals	Mice, mixed strains, ages E15.5 to 6 months, male and female. Cmya5Δ/Δ (C57BL/6NJ-Cmya5em1(IMPC)J/Mmja; Stock no. 032826, Jackson Laboratory); RosaCas9GFP/Cas9GFP (Gt(ROSA)26Sortm1(CAG-cas9*,-EGFP)Fezh; Stock No. 026175, Jackson Laboratory);Ryr2-GFP knockin (129sve, gift from Dr. Wayne Chen, University of Calgary). Descriptions of mice used for experiments can be found in the relevant figure legends and Methods. All animal experiments were performed under protocols approved by the Boston Children’s Hospital Institutional Animal Care and Use Committee.
Wild animals	No wild animals were used in the study.
Field-collected samples	No field collected samples were used in the study.
Ethics oversight	Boston Children's Hospital (BCH) Institutional Animal Care and Use Committee (IACUC)

Note that full information on the approval of the study protocol must also be provided in the manuscript.
